# ExCNVSS: A Noise-Robust Method for Copy Number Variation Detection in Whole Exome Sequencing Data

**DOI:** 10.1155/2017/9631282

**Published:** 2017-06-18

**Authors:** Jinhwa Kong, Jaemoon Shin, Jungim Won, Keonbae Lee, Unjoo Lee, Jeehee Yoon

**Affiliations:** ^1^Department of Computer Engineering, Hallym University, Chuncheon, Republic of Korea; ^2^Smart Computing Lab, Hallym University, Chuncheon, Republic of Korea; ^3^Department of Electronic Engineering, Kyonggi University, Suwon, Republic of Korea; ^4^Department of Electronic Engineering, Hallym University, Chuncheon, Republic of Korea

## Abstract

Copy number variations (CNVs) are structural variants associated with human diseases. Recent studies verified that disease-related genes are based on the extraction of rare de novo and transmitted CNVs from exome sequencing data. The need for more efficient and accurate methods has increased, which still remains a challenging problem due to coverage biases, as well as the sparse, small-sized, and noncontinuous nature of exome sequencing. In this study, we developed a new CNV detection method, ExCNVSS, based on read coverage depth evaluation and scale-space filtering to resolve these problems. We also developed the method ExCNVSS_noRatio, which is a version of ExCNVSS, for applying to cases with an input of test data only without the need to consider the availability of a matched control. To evaluate the performance of our method, we tested it with 11 different simulated data sets and 10 real HapMap samples' data. The results demonstrated that ExCNVSS outperformed three other state-of-the-art methods and that our method corrected for coverage biases and detected all-sized CNVs even without matched control data.

## 1. Introduction

Recent technological advances in next-generation sequencing (NGS) and massively accumulated exome sequencing data highlight the need to detect disease-related genes and genetic variations from exome sequencing. The analysis of exome sequencing data became available even in small-scale laboratories due to its low-level memory requirement and decreased computational complexity compared to whole genome sequencing data. Furthermore, recent developments in many web-based and/or cloud-based pipelines of exome sequencing data analysis facilitate analyses, such as preprocessing, alignment processing, variant detection, and functional study, especially in small-scale laboratories [1, 2].

However, these pipelines are restricted to the extraction of simple variants, such as SNPs and short indels, which are not suitable for detecting structural variants (SV), such as copy number variations (CNVs) and large indels. A CNV is defined as a DNA segment of 50 bp or larger and present at a variable copy number in comparison with a reference genome. A CNV is an important variant associated with human diseases such as autism, intellectual disability, epilepsy, schizophrenia, obesity, and cancer [3–6]. Specifically, researchers verified disease-causing genes based on the extraction of rare, de novo, and transmitted CNVs from exome sequencing data [7–9].

However, exome-based CNV detection still remains a challenging problem due to two obstacles: one is the presence of coverage biases introduced by the capture and sequencing of exomes and the other is the sparse, small size, and noncontinuous nature of target regions [10]. There are publically available CNV detection methods based on read depth approaches, including ExomeCNV [11], Contra [12], CoNIFER [13], XHMM [14], and Excavator [15]. Each of these methods implements key strategies to mitigate coverage biases caused by the capture and sequencing of exomes. ExomeCNV involves a modeling method using the Geary-Hinkley transformation to obtain normally distributed read coverage data. Contra adopts a normalization method that includes the use of base-level log-ratios and corrects for an imbalanced library size. Both CoNIFER and XHMM combine read coverage data with singular value decomposition (SVD) and principal component analysis (PCA) methods to identify and remove experimental noise. Excavator adopts a median normalization procedure to reduce systematic biases due to GC content, mappability, and exon size. While some of these methods reduce systematic biases in test data by efficiently utilizing many samples, they may have a limited application only in sequencing experiments dealing with a large number of samples. CoNIFER and XHMM require many samples at once in order to normalize the test data by SVD and PCA procedures. The baseline control suggested by Contra also requires many samples to generate a pooled model.

To detect the boundaries of variant regions, some of these CNV detection methods adopt a simple or modified circular binary segmentation algorithm [16], which usually performs well for subdividing a continuous region. However, this may result in missing larger or smaller variants due to sparsely targeted regions in exome sequencing data [15].

To overcome the obstacles presented by conventional methods, we developed a new CNV detection method, ExCNVSS, based on read coverage depth evaluation and scale-space filtering [17]. Our key strategies include correcting coverage biases introduced by capture and sequencing through read coverage depth evaluation and consideration for the sparse, small size, and noncontinuous nature of target regions through the multiresolution system of scale-space filtering. This enables the detection of different types and the exact location of CNVs of all sizes. Furthermore, ExCNVSS_noRatio, a version of ExCNVSS developed with the intention of applying it to the case of only the input of test data and without using control data, can detect all-sized copy number gains and losses for concatenated, arbitrary-sized exonic regions even when a matched control is not available.

Our method can be summarized as follows: (1) It extracts base-level read coverage depth within each targeted exonic region from the read alignment data and merges them to generate concatenated base-level read coverage data. (2) To reduce the coverage bias effect, base-level read coverage data are normalized by our four-step normalization protocol. In each step, target exon read coverage data are considered to be evaluated from test data only or from the ratio of test and control data, according to the contents of the input, test data only, or both test and control data. (3) The scale-space filtering is then applied to normalized base-level read coverage data using a Gaussian convolution for various scales according to a given scaling parameter. By differentiating the scale-space filtered data twice and then finding zero-crossing points of the second derivatives, inflection points of the scale-space filtered data are calculated per scale. (4) Finally, the types and exact locations of CNVs of test data are obtained by using parametric baselines, which are evaluated from the normalized base-level coverage data, and by analyzing the finger print map, which is the collection of contours of the zero-crossing points for various scales.

We carried out simulation experiments to assess the performance of ExCNVSS and to extract the optimal values of parametric baselines from the results. The performance assessment of ExCNVSS was obtained by evaluating the false negative rate (FNR) and false positive rate (FPR) on the basis of the number of detected target-level CNV regions in transcript coordinates. The performance of ExCNVSS was then compared with conventional methods. In addition, the performance of ExCNVSS was validated by experiments with 10 individual HapMap samples using optimal parametric baselines. The results of the experiments showed a reasonable trade-off between FNR and FPR, even when an artificial data set was used as a pseudo-control, which showed that ExCNVSS could precisely detect CNVs of various types and sizes.

## 2. Materials and Methods

**Figure 1:**
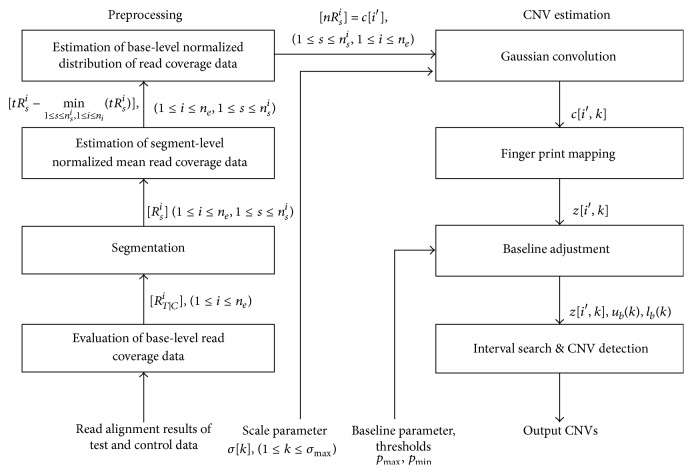
The flowchart of our method. It includes two procedures: data preprocessing and CNV estimation. The data preprocessing procedure included a four-step normalization protocol. The CNV estimation procedure included a Gaussian convolution, finger print mapping, baseline adjustment, interval search, and CNV detection.

Figure 1 shows the flowchart of the overall process of our method. It includes two procedures: data preprocessing and CNV estimation. A new, four-step normalization protocol was implemented for the data preprocessing procedure. The scale-space filtering, which is consisted of Gaussian convolution, finger print mapping, baseline adjustment, interval search, and CNV detection, was applied for the CNV estimation procedure [18].

### 2.1. Preprocessing Data

The normalization protocol of the preprocessing procedure implemented consisted of four steps: evaluation of base-level read coverage data, segmentation, estimation of segment-level normalized mean read coverage data, and estimation of base-level normalized distribution of read coverage data in order to minimize the effect of the sources of variation, such as GC content bias [19], library size effect [20], and exon edge bias [21]. In each step, the read coverage data were considered to be evaluated from test data only or from the ratio of test and control data, according to the contents of the input, test data only, or both test and control data. The details of each step are described in the following subsections in which the case of the input with respect to test and control data is considered.

#### 2.1.1. Evaluation of Base-Level Read Coverage Data

Read coverage data *R*_*T*_ and *R*_*C*_ were extracted from input data *T*and *C*, which were read alignment results of test and control genomic sequencing data, respectively. The concatenated target exon read coverage data *R*_*T*_^*e*^ = *R*_*T*_^1^*R*_*T*_^2^ ⋯ *R*_*T*_^*n*_*e*_^ and *R*_*C*_^*e*^ = *R*_*C*_^1^*R*_*C*_^2^ ⋯ *R*_*C*_^*n*_*e*_^ were extracted from the read coverage data *R*_*T*_ and *R*_*C*_, respectively, where *R*_*T*_^*i*^ = *t*_1_^*i*^*t*_2_^*i*^ ⋯ *t*_*n*_*i*__^*i*^ and *R*_*C*_^*i*^ = *c*_1_^*i*^*c*_2_^*i*^ ⋯ *c*_*n*_*i*__^*i*^ are the *i*th target exon read coverage data with length *n*_*i*_ of test and control data, respectively, and *n*_*e*_ is the total number of target exons. Then, the base-level read coverage data were obtained by evaluating the target exon read coverage ratio data *R*_*T*∣*C*_^*e*^ = *R*_*T*∣*C*_^1^*R*_*T*∣*C*_^2^ ⋯ *R*_*T*∣*C*_^*n*_*e*_^, where *R*_*T*∣*C*_^*i*^ is the sequence of the ratio [*r*_*j*_^*i*^] = [*t*_*j*_^*i*^/*c*_*j*_^*i*^], 1 ≤ *j* ≤ *n*_*i*_, of the *i*th target exon read coverage data *R*_*T*_^*i*^ = *t*_1_^*i*^*t*_2_^*i*^ ⋯ *t*_*n*_*i*__^*i*^ and *R*_*C*_^*i*^ = *c*_1_^*i*^*c*_2_^*i*^ ⋯ *c*_*n*_*i*__^*i*^, multiplied by the parameter *ω* for correcting the imbalanced library size effect between test *R*_*T*_ and control *R*_*C*_ read coverage data. The parameter *ω* is the ratio *mR*_*C*_^*e*^/*mR*_*T*_^*e*^ of the total sums *mR*_*T*_^*e*^ = ∑_*i*_^*n*_*e*_^∑_*j*_^*n*_*i*_^*t*_*j*_^*i*^ and *mR*_*C*_^*e*^ = ∑_*i*_^*n*_*e*_^∑_*j*_^*n*_*i*_^*c*_*j*_^*i*^ of the test and control read coverage data in all the target exons. In the estimation of the sequence of the ratio [*r*_*j*_^*i*^], 1 ≤ *j* ≤ *n*_*i*_, the cases of the read coverage data with values of nearly zero are considered as follows, where *ε* represents a very small nonnegative number which is here set to be 10^−3^:

(1) $$\begin{array}{c} r_{j}^{i}=\left\{\begin{array}{cc} 0, & t_{j}^{i}<\varepsilon \\ 1, & \left(t_{j}^{i}<\varepsilon \right)\&\left(c_{j}^{i}<\varepsilon \right)\\ \frac{t_{j}^{i}}{c_{j}^{i}}, & \text{others}. \end{array}\right. \end{array}$$

#### 2.1.2. Segmentation

The sequence *R*_*T*∣*C*_^*i*^ = [*ω* · *r*_*j*_^*i*^], 1 ≤ *i* ≤ *n*_*e*_,  ⌊mod (*n*_*i*_, *b*_*s*_)/2⌋ < *j* ≤ ⌊mod (*n*_*i*_, *b*_*s*_)/2⌋ + *b*_*s*_ × ⌊*n*_*i*_/*b*_*s*_⌋ of each target exon is partitioned into *n*_*s*_^*i*^ = ⌊*n*_*i*_/*b*_*s*_⌋ segment sequence [*R*_*s*_^*i*^], 1 ≤ *s* ≤ *n*_*s*_^*i*^, with *b*_*s*_ equal size starting from *j* = ⌊mod (*n*_*i*_, *b*_*s*_)/2⌋ + 1, where the remnant sequences between 1 ≤ *j* ≤ ⌊mod (*n*_*i*_, *b*_*s*_)/2⌋ and ⌊mod (*n*_*i*_, *b*_*s*_)/2⌋ + *b*_*s*_ × ⌊*n*_*i*_/*b*_*s*_⌋ < *j* ≤ *n*_*i*_ are neglected.

#### 2.1.3. Estimation of Segment-Level Normalized Mean Read Coverage Data

The mean *mR*_*s*_^*i*^ = ∑_*j*∈*s*_*ω* · *r*_*j*_^*i*^/*b*_*s*_, 1 ≤ *s* ≤ *n*_*s*_^*i*^, 1 ≤ *i* ≤ *n*_*e*_ of each segment was adjusted to be the normalized mean $tR_{s}^{i}=(mR_{s}^{i}-{mean}_{1\leq s\leq n_{s}^{i},1\leq i\leq n_{e}}(mR_{s}^{i}))/({std}_{1\leq s\leq n_{s}^{i},1\leq i\leq n_{e}}(mR_{s}^{i})/\sqrt{n_{e}\times n_{s}^{i}})$, 1 ≤ *s* ≤ *n*_*s*_^*i*^, 1 ≤ *i* ≤ *n*_*e*_, by using its *t*-score, where mean_1≤*s*≤*n*_*s*_^*i*^,1≤*i*≤*n*_*e*__(*mR*_*s*_^*i*^) and std_1≤*s*≤*n*_*s*_^*i*^,1≤*i*≤*n*_*e*__(*mR*_*s*_^*i*^) are the mean and standard deviation of the means of each segment in all target exons. Then, the normalized mean *tR*_*s*_^*i*^, 1 ≤ *s* ≤ *n*_*s*_^*i*^, 1 ≤ *i* ≤ *n*_*e*_, of each segment was shifted by the minimum value min_1≤*s*≤*n*_*s*_^*i*^,1≤*i*≤*n*_*i*__⁡(*tR*_*s*_^*i*^) of the normalized means in order to ensure that the mean of each segment was not to be less than zero.

#### 2.1.4. Estimation of the Base-Level Normalized Distribution of Read Coverage Data

The sequence *R*_*s*_^*i*^, 1 ≤ *s* ≤ *n*_*s*_^*i*^, 1 ≤ *i* ≤ *n*_*e*_, of each segment of the *i*th target exon read coverage ratio data was readjusted into *nR*_*s*_^*i*^, 1 ≤ *s* ≤ *n*_*s*_^*i*^, 1 ≤ *i* ≤ *n*_*e*_, to be normally distributed within the segment based on the normalized mean *tR*_*s*_^*i*^ and the standard deviation std_∈*s*_(*R*_*s*_^*i*^) of the segment.

### 2.2. Copy Number Estimation

The CNV estimation procedure included five steps: Gaussian convolution, finger print mapping, baseline adjustment, interval search, and CNV detection as described in our previous work [18]. Some changes were necessary in the steps of baseline adjustment and CNV detection to reduce the effect of the sources of variation. Therefore, descriptions of the CNV estimation procedure mainly concerned changed parts in the steps of baseline adjustment and CNV detection in this section.

In the Gaussian convolution step, the sequence *c*[*i*′] = [*nR*_*s*_^*i*^] = *c*_1_*c*_2_ ⋯ *c*_*n*_, *n* = *b*_*s*_ × ∑_*i*_^*n*_*e*_^*n*_*s*_^*i*^, of readjusted target exon read coverage ratio data obtained in the preprocessing procedure was decomposed into *l* layers by Gaussian convolution with increasing *σ* as in the following equation:

(2) ci′,kci′∗gj′,σk=∑j=−mmci′−j′1σk2πe−j′2/2σk2,

where *c*[*i*′, *k*] is the scale-space image of *c*[*i*′], *k*  (0 ≤ *k* ≤ *l* − 1), representing the index of the layer of the scale-space image, *σ*_*k*_ is the value of the scale parameter at layer *k*, and *m* is the window size of the Gaussian kernel *g*[*j*′, *σ*_*k*_], which is set to *m* = 3*σ*_*k*_. The scale parameter *σ*_*k*_ is the standard deviation of the Gaussian kernel *g*[*j*′, *σ*_*k*_] and is set to *σ*_*k*_ = 10^2^ × (1.1)^*k*^ considering the range of detectable CNV size and time complexity. Here, we obtained the scale-space image *c*[*i*′, *k*] of *c*[*i*′] by applying a discrete Fourier transform in the frequency domain to reduce the computational complexity. Let *C*[*w*] and *G*[*w*, *k*] = *e*^−*w*^2^*σ*_*k*_^2^/2^ be the discrete Fourier transform of *c*[*i*′] and *g*[*j*′, *σ*_*k*_], respectively. The scale-space image was then obtained by *c*[*i*′, *k*] = *ℑ*^−1^{*G*[*w*, *k*]*C*[*w*]}, where *ℑ*^−1^ is the inverse discrete Fourier transform operator. Then, the zero-crossing points of the second-order derivatives of the scale-space image *c*[*i*′, *k*] were searched for in each layer *k*(0 ≤ *k* ≤ *l* − 1) in the step of fingerprint mapping. Here, the second derivative *c*′′[*i*′, *k*] of *c*[*i*′, *k*] was approximated by the second-order difference, *c*′′[*i*′, *k*] ≈ *c*[*i*′ + 1, *k*] − 2*c*[*i*′, *k*] + *c*[*i*′ − 1, *k*]. A zero-crossing signal *z*[*i*′, *k*] was defined as follows:

(3) zi′,k=+1,c′′i′+1,k>0&c′′i′−1,k<0−1,c′′i′+1,k<0&c′′i′−1,k>00,others,

where the condition (*c*′′[*i*′ + 1, *k*] > 0)&(*c*′′[*i*′ − 1, *k*] < 0) represents the zero-crossing point *i*′ at which *c*′′[*i*′, *k*] crosses zero from minus to plus and the condition (*c*′′[*i*′ + 1, *k*] < 0)&(*c*′′[*i*′ − 1, *k*] > 0) from plus to minus. Next, in the baseline adjustment step, two parametric baselines, *u*_*b*_(*k*) and *l*_*b*_(*k*), were calculated for each layer that had more than two nonzero elements in the zero-crossing signal using an empirical cumulative distribution function of the scale-space image, *c*[*i*′, *k*]. The parametric baseline *u*_*b*_(*k*) was estimated to be the lowest value of the scale-space image among those ranked within a given threshold *p*_max_ from the top at layer *k*. Similarly, the parametric baseline *l*_*b*_(*k*) was estimated to be the highest value of the scale-space image among those ranked within a given threshold *p*_min_ from the bottom at layer *k*, where the threshold *p*_min_ was especially decided considering the portion of the test read coverage data with a value of zero. In the interval search step, intervals were searched from the zero-crossing signal *z*[*i*′, *k*] using the parametric baselines *u*_*b*_(*k*) and *l*_*b*_(*k*) for each layer. The *m*th interval [*l*_*m*,*k*_, *u*_*m*,*k*_] at layer *k* was defined as a closed interval {*i*′∣*l*_*m*,*k*_ ≤ *i*′ ≤ *u*_*m*,*k*_} in the position index *i*′ of the zero-crossing signal *z*[*i*′, *k*], which is a set of the position indices of *z*[*i*′, *k*] between *l*_*m*,*k*_ and *u*_*m*,*k*_ inclusive, satisfying the following three conditions to be a putative CNV region. First, the interval [*l*_*m*,*k*_, *u*_*m*,*k*_] does not include position indices corresponding to all regions of CNVs already declared at layers above the layer *k*. Second, *z*[*l*_*m*,*k*_, *k*] · *z*[*u*_*m*,*k*_, *k*] < 0 and *z*[*i*′, *k*] = 0 for all the position indices between *l*_*m*,*k*_ and *u*_*m*,*k*_. Third, the average ∑_*i*′=*l*_*m*,*k*__^*u*_*m*,*k*_^*c*[*i*′, *k*]/(*u*_*m*,*k*_ − *l*_*m*,*k*_ + 1) of the scale-space image on the position indices between *l*_*m*,*k*_ and *u*_*m*,*k*_ inclusive is beyond the given parametric baselines, *u*_*b*_(*k*) or *l*_*b*_(*k*). Once we had the *m*th interval [*l*_*m*,*k*_, *u*_*m*,*k*_] as a putative CNV region, we traced the zero-crossing signal *z*[*i*′, *k*] from the positions *l*_*m*,*k*_ and *u*_*m*,*k*_ at layer *k* until we obtained the corresponding positions *l*_*m*,*k*_′ and *u*_*m*,*k*_′, respectively, bounded at layer *k* = 0, where the closed interval [*l*_*m*,*k*_′, *u*_*m*,*k*_′] = {*i*′∣*l*_*m*,*k*_′ ≤ *i*′ ≤ *u*_*m*,*k*_′} is to be declared as a CNV. Finally, the CNV detection step was preceded by searching for intervals from the top layer to the bottom layer sequentially. When searching for intervals at layer *k*, the sum of sets ⋃_*s*′=*k*+1_^*k*_max_^⋃_*m*=1_^*m*_max,*s*′_^[*l*_*m*,*s*′_′, *u*_*m*,*s*′_′] corresponding to all the regions of CNVs already declared at the upper layers from *k* + 1 to *k*_max_ were excluded, where *m*_max,*s*′_ is the total number of CNVs detected at layer *s*′, as described in a previous work [18]. The type and localization of a CNV were determined by using the results of the interval search. An interval [*l*_*m*,*k*_, *u*_*m*,*k*_] identifies the region where a statistically significant variation occurred on the input sequence and a CNV gain or loss was to be detected. That is, a CNV gain or loss was identified if the average ∑_*i*′=*l*_*m*,*k*__^*u*_*m*,*k*_^*c*[*i*′, *k*]/(*u*_*m*,*k*_ − *l*_*m*,*k*_ + 1) of a scale-space image in the interval was above *u*_*b*_(*k*) or below *l*_*b*_(*k*), respectively. Then, the localization of a CNV was defined by tracing to the corresponding region [*l*_*m*,*k*_′, *u*_*m*,*k*_′] as the layer *k* converges to zero.

### 2.3. Materials

TargetedSim [http://sourceforge.net/projects/targetedsim/] is a simulation tool that creates paired-end reads from targeted regions in a chromosome and can also simulate gains or losses of CNVs at random locations within targeted regions. For generating a simulated exome read data set, we used the TargetedSim tool, which has been developed by the Contra project group [12]. Test and control data were simulated as Illumina paired-end reads using chromosome 1 of the human reference assembly (hg19). The read length and median insert size of the simulated data were 36 bp and 200 bp, respectively. The simulated data covered 21,881 target regions (a total length of 5,256,986 bp, average length of 240 bp, minimum length of 115 bp, and maximum length of 8,551 bp) in chromosome 1, which are the same data used by the Agilent SureSelect Human All Exon 50 Mb V3 capture platform [https://earray.chem.agilent.com/suredesign/]. We generated 11 test data sets, each of which contained approximately 20 to 30 CNV regions, corresponding to approximately 70–100 target regions of an appropriate size (an average length of 222 bp, a minimum length of 120 bp, and a maximum length of 8,260 bp in the transcript coordinate). We aligned test and control read data to the human reference assembly using BWA [http://bio-bwa.sourceforge.net/] and obtained test and control BAM files with an average coverage level of 40x, respectively, which is assumed to be the bottom limit of a reasonable amount of sequence for variants calling.

The exome sequencing data downloaded from the 1000 Genome Project website (http://www.1000genomes.org) were used for the experiment with real human data. The downloaded data were BAM format files of 10 HapMap samples: NA12843 (47x), NA12842 (182.6x), NA12748 (49.5x), NA12718 (102.9x), NA12275 (86.9x), NA12273 (77.2x), NA12272 (92.6x), NA11843 (50.9x), NA10847 (99.2x), and NA06984 (54x), each of which is a member of the Utah residents (CEU) population and was sequenced in the same BI genome center and captured using the same assay (Agilent SureSelect Human All Exon V2). One individual sample of NA19152 (101.6x) was also downloaded for use as a control data set, which is a member of the Yorba (YRI) population, sequenced and captured by using the same technology as the test data sets. The capture platform covered 20,258 target regions (a total length of 4,775,342 bp, average length of 235 bp, a minimum length of 115 bp, and a maximum length of 8,551 bp) in chromosome 1.

As the downloaded 10 germline data sets were generated without the availability of matched control data sets, a pseudo-control data set had to be created to serve as the control. There are two methods that have been used to generate a control sample data: one derives a matched control data set from a pool of other samples by averaging the depth of coverage of each exon across all exomes; the other uses a specific and different germline sample as the control. In general, generating a pooled sample is tedious and time-consuming work that entails preprocessing tens or hundreds of samples, which are captured and sequenced by the same platform. When a specific individual sample is used as a pseudo-control, the selection is made carefully such that (1) the pseudo-control sample is a member of other populations having a different background (not genetically related), (2) it is captured using the same probe set and capture method and sequenced in the same manner as the test samples, and (3) it has the same gender as the test samples.

However, even if a pooled sample is generated from many well-selected independent samples or a specific sample is selected from unrelated individuals, such as from a different population, we cannot ascertain that this pseudo-control data actually has an average genomic normal copy number of 2 and does not share common CNV regions with the test sample data [11].

The pseudo-control should capture the technical variation of a platform, but not CNV variations in the test sample. With these considerations, we propose using an artificially simulated data as pseudo-control data. Currently, there have been various simulation methods that generate reads by emphasizing different characteristics of real sequencing data for various applications. Wessim [22] particularly aims for a real exome sequencing simulation. As effective pseudo-control read data, we adopted a simulated exome data by Wessim. Wessim emulates conventional exome capture technologies, such as Agilent's SureSelect and Roche/NimbleGen's SeqCap, and generates realistic synthetic exome sequencing data, in which fragment length and GC content are rigorously considered to reproduce accurate coverage biases. We aligned the pseudo-control read data to a human reference assembly using BWA and generated a BAM file with an average coverage level of 40x.

The BAM file of each of the test and control samples was processed, sorted, and filtered with SAMtools [http://samtools.sourceforge.net/]. After removing PCR duplicate reads with MarkDuplicates of Picard [http://picard.sourceforge.net], local realignment around indel was performed using the RealignerTargetCreator and IndelRealigner of GATK [https://software.broadinstitute.org/gatk/].

The performance of ExCNVSS was assessed by estimating the FNR and FPR on the basis of the number of detected target-level CNV regions in transcript coordinates. Each region was considered validated if an algorithm called for more than 30% of synthetic or known CNV regions. ExCNVSS was compared with three conventional CNV detection methods: ExomeCNV, Contra, and Excavator. Furthermore, the performances of all four methods were assessed and compared with ExCNVSS_noRatio.

The experiments were carried out in Windows 7 and CentOS 6.2 on an Intel Core i7 3.5 GHz CPU with 32 GB of main memory and a 2 TB hard drive. The programming language used for the development of ExCNVSS was MATLAB.

## 3. Results and Discussion

### 3.1. Experiments with Simulated Data

The first experiment was carried out to assess the performance of ExCNVSS according to various values of the threshold values *p*_max_ and *p*_min_, which were used for determining the parametric baselines. Performance was assessed by estimating FNRs and FPRs on the basis of the number of detected target-level CNV regions. The experiments for each threshold value were performed with 11 different simulated data sets, the results of which were averaged for the assessment. The overall FNRs and FPRs were in the range of 10.93–13.76% and 3.11–62.21%, respectively, for various threshold values (*p*_max_ and *p*_min_). The best performance was obtained at threshold values of *p*_max_ of 0.9875 and *p*_min_ of 0.0125, where the values of FNR and FPR were 13.76% and 3.11%, respectively. Therefore, the threshold values *p*_max_ of 0.9875 and *p*_min_ of 0.0125 were used as defaults for ExCNVSS. Similarly, ExCNVSS_noRatio showed FNRs and FPRs in the range of 26.73–29.33% and 5.94–46.66%, respectively. The best performance of ExCNVSS_noRatio was obtained at *p*_max_ of 0.9875 and *p*_min_ of 0.04, where the values of FNR and FPR were 26.73% and 5.94%, respectively. Therefore, threshold values of *p*_max_ of 0.9875 and *p*_min_ of 0.04 were used as defaults for ExCNVSS_noRatio.

The performance of ExCNVSS was compared with that of ExCNVSS_noRatio, Contra, Excavator, and ExomeCNV on 11 simulated data sets, where various parameters of each method were determined according to the instructions in each manual. The parameters variable in Contra, Excavator, and ExomeCNV are as follows: Contra (numBin, minReadDepth, minNBases, pval, and nomultimapped); Excavator (*ω*, *θ*, *d*Norm, *c*, seg, *u*, and *l*); ExomeCNV (coverage.cutoff, admix, sdundo, alpha, min.spec, and min.sens). We calculated the performance in order to check the increase in FPRs and the change in FNRs while changing values of pval (*p* value threshold for filtering) for Contra, *θ* (baseline probability) for Excavator, and min.spec (desired minimum specificity) for ExomeCNV, which seemed to be directly related to FPR.

The overall FNR of Contra was between 23.11% and 33.88% and the FPR between 0.02% and 93.83% for various pval values. Contra achieved an average FNR of 33.88% and FPR of 0.02% with its default setting. The overall FNR for Excavator was between 27.99% and 52.60% and the FPR between 0.23% and 82.11% for various *θ* values. Excavator showed an average FNR of 52.60% and FPR of 0.23% with its default parameter settings. The overall FNR for ExomeCNV was between 45.52% and 88.69%, and the FPR was between 0.02% and 94.02% for various min.spec values. ExomeCNV showed an average FNR of 88.69% and FPR of 0.02% with its default setting.

**Figure 2:**
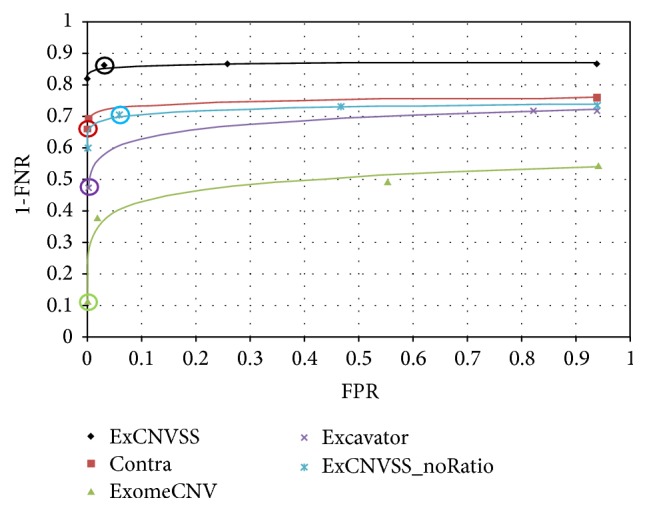
The ROC curves of the five methods. FNRs and FPRs were calculated on 11 simulated data sets at different threshold levels, and ROC curves were generated on the basis of averaged values. The circled symbol on each curve represents the performance of each method using default parameters.

Figure 2 presents the receiver operation characteristic (ROC) curves of ExCNVSS, ExCNVSS_noRatio, Contra, Excavator, and ExomeCNV for comparison. As shown in the ROC curves, the performance of ExCNVSS was better than those of the other four methods. The conventional methods, including Contra, Excavator, and ExomeCNV, were very conservative in calling a region significant, resulting in high FNRs and low FPRs with default parameter settings. Although some parameters can be varied to relax the specificity for these methods, remarkable improvements have not been observed in FNRs. However, ExCNVSS_noRatio provided a good performance in FNR with little increase in FPR, even though control data to compensate for inherent coverage biases were not applied. These results suggest that both ExCNVSS and ExCNVSS_noRatio can be very robust in error-prone environments, resulting in a good performance even at relatively low-level coverage data.

The second experiment was carried out to assess the performance of ExCNVSS with respect to the size of CNVs. The size of a target region in most exome capture platforms is typically small and approximately 90% of target regions are <300 bp in length. For the experiment, we simulated 861 loss and gain target regions (minimum length of 120 bp, maximum length of 8,260 bp, and an average length of 222 bp), including single exon losses and gains, as well as variations spanning multiple exons in the test data sets. We also generated control data sets with no CNVs and the same mean coverage (40x) as the test data sets.

Table 1 shows the performance of methods on simulated data sets, representing the total number of correctly detected instances of small (100–159 bp), medium (160–299 bp), and large (300–8260 bp) variants, along with the fraction of gain/loss regions of each in parentheses. The second and third columns for each method represent false negative and false positive rates and the range of detected gain/loss region sizes (min/max), respectively.

The results show that ExCNVSS is superior to the other four methods in terms of detecting CNVs of various sizes. As ExCNVSS detects larger CNVs at a higher scale and smaller CNVs at a lower scale, the FNR can be reduced in various CNV sizes compared to conventional methods using target-level log-ratio detection and segmentation. Additionally, compared with other methods, ExCNVSS detects more CNV loss regions, which may represent severe mutations in Mendelian diseases. It has been acknowledged that CNV losses are usually more harmful because a great deal of genetic information is missing, whereas CNV gains involve repeating nucleotide units.

ExCNVSS_noRatio showed a slightly lower performance in detecting larger CNVs than the small or medium-sized CNVs. This could be because biases may not be compensated sufficiently at large CNV regions by our segmentation and normalization method without control data. Contra achieves a good performance in detecting medium-sized CNVs, while the FNR increased in detecting smaller and larger CNVs. As previously mentioned, Contra, Excavator, and ExomeCNV are conservative in calling a region significant and they show relatively high FNRs and low FPRs with default parameter settings. We can deduce that ExCNVSS and ExCNVSS_noRatio are effective methods in detecting CNVs of various sizes by reducing the inherent noise in exome read coverage data.

### 3.2. Experiments with HapMap Samples

The downloaded BAM files of 10 HapMap samples were used for experiments with real human data. The performance assessment was accomplished by evaluating the FNR and FPR on the basis of the Phase 3 variant list of the 1000 Genome project released in 2014. Each region was considered validated if the algorithm called for more than 30% of the known CNV region profiled in the Phase 3 variant list. However, it should be noted that a true gold standard CNV list for these HapMap samples is still not available, and this list does not have 100% sensitivity and specificity [23].

As previously mentioned, ExCNVSS, Excavator, Contra, and ExomeCNV require two input data samples, test and control, to identify CNV variants. In this real data experiment, two different types of pseudo-control data were used: one was an artificial data set that simulates realistic synthetic exome sequencing data, and the other was a specific sample data set that was selected from unrelated individuals, such as from a different population.

In the first experiment, we used an artificial exome data generated by Wessim [http://sak042.github.io/Wessim/]. Wessim provides two distinct approaches for exome read generation: ideal target approach and probe hybridization approach. Using probe hybridization approach is recommended when the probe sequence is available; it is much more realistic and recovers the statistics of real data with default parameter setting.  Table 2 describes a quantitative analysis of experimental results on the whole region of chromosome 1 of the 10 HapMap samples, in which the performance of ExCNVSS was compared with those of ExCNVSS_noRatio, ExomeCNV, Contra, and Excavator. Here, ExomeCNV, Contra, and Excavator adopted the same Wessim data set as the control.

In general, selecting the optimal parameter matching the characteristics of the data is crucial in increasing an algorithm's performance. However, finding the optimal parameters of each algorithm requires the exact understanding of the mathematical model derived from the interaction between multiple parameters, which is classified as an extremely difficult and cautious operation.

Therefore, we referred to the part of the evaluation of performances using HapMap samples in the original paper of each algorithm and used the values of the parameters as the optimal parameters of each algorithm for our real data experiments using HapMap samples. In paper [15] covering Excavator, parameters (*ω* = 0.1, *θ* = 10^−4^, *d*Norm = 10^5^, and *c* = 1) were used for the analysis of 20 HapMap samples, and in paper [12] covering Contra, parameter (*p*val = 0.01) was used for the analysis of 5 HapMap samples for their evaluation of performance test results. Only in the case of ExomeCNV, the parameters used for the analysis of HapMap samples were not specified [11]. As a result, in the cases of Excavator and Contra the parameter values given in the original paper were used as the optimal parameters for our own experiment, and in the case of ExomeCNV the default parameters used in the performance test in previous papers were used as the optimal parameters for our own experiment.

Table 2 shows the results of the performance evaluation of each algorithm using real data with the optimal parameters. In Table 2, the first column for each method represents the total number of correctly detected instances and the fraction of gain/loss regions is given in parentheses. The second column represents false negative and false positive rates, respectively.

In the 10 HapMap samples, ExCNVSS obtained the best results for FNR, followed by ExomeCNV, Excavator, ExCNVSS_noRatio, and Contra. Contra was the best for FPR, followed by Excavator, ExCNVSS, ExCNVSS_noRatio, and ExomeCNV. Among these five methods, both ExCNVSS and Excavator showed the best performances. The overall FNRs for ExCNVSS and Excavator were between 25.64% and 45.54% and between 33.68% and 54.81%, respectively. The FPRs for ExCNVSS and Excavator were between 10.45% and 13.21% and between 4.43% and 6.10%, respectively. However, Excavator produced poor results in identifying CNV loss regions due to only a small number of CNV events being detected. ExomeCNV obtained a relatively good performance in FNR since it returned a large number of instances of CNVs. In contrast, Contra showed poor performance in FNR, since it returned only a small number of instances of CNVs. Collectively, ExCNVSS showed a reasonable trade-off between FNR and FPR, which efficiently detected CNVs of various types and sizes.

In the second experiment, we used the exome read data from an individual sample of NA19152 (a member of the YRI population) as a control data set, which was also used as a control for the analysis of HapMap samples in paper [15].  Table 3 describes the experimental results on HapMap samples, each of which is a member of the CEU population with the optimal parameters. In the 10 HapMap samples, all methods gave poor results, with the exception of ExCNVSS_noRatio. Even ExCNVSS and Excavator gave poor performance in FNR while preserving similar performance in FPR compared with the first experiment. However, although no control data were used, ExCNVSS_noRatio showed a better performance than the other methods.

From the results, we can see that, even through the efforts to select a proper pseudo-control sample from the other individual samples, we could not remove the biases introduced by capture and sequencing at all. The adoption of the control data standards is a crucial process in variants calling, as it may help to manage the inherent noise of the test data, affecting the overall performances of the methods. These results show that a well-made simulated data set can be used as a good alternative control to reduce coverage biases of the test data, compared to using real data. Furthermore, ExCNVSS_noRatio can be an alternative to ExCNVSS in the absence of proper matched control data.

## 4. Conclusions

As advanced NGS technologies produce a large number of short reads at lower costs and increased speeds that accumulate exome sequencing data, the need to detect even small disease-related genetic variations directly from exome sequencing is expected to drastically increase. We have developed an exon-based CNV detection method using read coverage depth evaluation and scale-space filtering. Our method corrects coverage biases and considers the sparse, small size, and noncontinuous nature of target regions. We tested the method on both simulated and real data, and the results show that the method can be applied to relatively low-level coverage data with practical specificity and sensitivity. We have also developed a method that can be applied to cases of input data only, and the results show that the method can detect all-sized CNV gains and losses for concatenated arbitrary-sized exonic regions, even when a matched control is not available.

The performances of our methods show excellent FNRs and relatively fair FPRs compared to conventional methods. Furthermore, the performance of our methods show the superiority of detecting CNVs of various sizes, with good values of FNRs and acceptable values of FPRs. Specially in the assessment using 10 real HapMap samples' data, one of our methods showed the best performance in FNRs and a fairly good performance in FPRs compared to conventional methods including ExomeCNV, Excavator, and Contra. This suggests that our method can reliably detect all-sized CNVs from sensitive exome sequencing data without considering the availability of a matched control. ExCNVSS and ExCNVSS_noRaio are freely accessible at http://dblab.hallym.ac.kr/ExCNVSS/.

## Figures and Tables

**Table 1:** CNV detection performances across variant sizes using simulated data sets. Each method was run with its default parameters.

Size of variants	100~159 bp	160~299 bp	300~8260 bp
Number of simulated instances (gain/loss)	438 (212/226)	430 (219/211)	93 (52/41)
Size of gain instances (min/max)	Gain (120 bp/151 bp)	Gain (184 bp/296 bp)	Gain (305 bp/8260 bp)
Size of loss instances (min/max)	Loss (120 bp/151 bp)	Loss (178 bp/299 bp)	Loss (301 bp/1561 bp)

ExCNVSS			
Number of correctly detected instances (gain/loss)	365 (164/201)	383 (192/191)	82 (45/37)
FNR/FPR (%)	16.7/2.7	10.9/2.2	12.1/6.3
Detected region size (bp)	Gain (120/151)	Gain (184/296)	Gain (305/8260)
(Min/max)	Loss (120/151)	Loss (178/299)	Loss (301/1561)
ExCNVSS_noRatio			
Number of correctly detected instances (gain/loss)	294 (105/189)	332 (141/191)	56 (18/38)
FNR/FPR (%)	32.8/6.4	22.8/4.3	42.0/9.5
Detected region size (bp)	Gain (120/151)	Gain (184/296)	Gain (305/8260)
(Min/max)	Loss (120/151)	Loss (178/299)	Loss (301/1561)
Excavator			
Number of correctly detected instances (gain/loss)	221 (100/121)	202 (94/108)	43 (26/17)
FNR/FPR (%)	50.3/0.1	53.4/0.1	52.8/0.8
Detected region size (bp)	Gain (120/151)	Gain (207/296)	Gain (305/871)
(Min/max)	Loss (120/151)	Loss (178/271)	Loss (359/603)
Contra			
Number of correctly detected instances (gain/loss)	247 (147/100)	371 (182/189)	44 (35/9)
FNR/FPR (%)	42.7/0.2	13.1/0.0	51.7/0.0
Detected region size (bp)	Gain (120/151)	Gain (184/296)	Gain (305/8260)
(Min/max)	Loss (120/151)	Loss (196/299)	Loss (303/1561)
ExomeCNV			
Number of correctly detected instances (gain/loss)	24 (24/0)	69 (69/0)	18 (17/1)
FNR/FPR (%)	94.3/0.0	84.3/0.0	78.8/0.0
Detected region size (bp)	Gain (120/151)	Gain (191/296)	Gain (305/8260)
(Min/max)	Loss (−/−)	Loss (−/−)	Loss (1561/1561)

**Table 2:** CNV detection performances using 10 real data sets. An artificial exome data set generated by Wessim was used as the control data set.

sample ID	ExCNVSS		ExCNVSS_noRatio		Excavator		Contra		ExomeCNV	
	Correctly detected instances (gain/loss)	FNR/FPR (%)	Correctly detected instances (gain/loss)	FNR/FPR (%)	Correctly detected instances (gain/loss)	FNR/FPR (%)	Correctly detected instances (gain/loss)	FNR/FPR (%)	Correctly detected instances (gain/loss)	FNR/FPR (%)
NA12843	99	26.67/	63	53.33/	61	54.81/	3	97.78/	100	25.93/
	(51/48)	12.39	(27/36)	14.99	(54/7)	4.43	(0/3)	0.05	(54/46)	43.35
NA12842	62	40.95/	39	62.86/	56	46.67/	0	98.10/	49	53.33/
	(50/12)	13.09	(25/14)	17.03	(56/0)	6.04	(0/2)	0.43	(49/0)	66.74
NA12748	59	41.58/	36	64.36/	63	37.62/	1	99.01/	56	44.55/
	(50/9)	13.21	(24/12)	14.47	(54/9)	5.46	(0/1)	0.04	(56/0)	55.38
NA12718	87	25.64/	76	35.04/	63	46.15/	7	94.02/	89	23.93/
	(49/38)	12.27	(31/45)	14.08	(54/9)	6.10	(0/7)	0.26	(54/35)	88.65
NA12275	57	41.84/	43	56.12/	56	42.86/	1	98.98/	56	42.86/
	(49/8)	11.53	(33/10)	13.74	(56/0)	6.09	(0/1)	0.27	(54/2)	91.48
NA12273	68	38.92/	40	64.60/	56	50.44/	4	96.46/	60	46.90/
	(54/14)	10.45	(27/13)	15.12	(56/0)	6.10	(0/4)	0.11	(49/11)	98.61
NA12272	58	41.41/	42	57.58/	64	35.35/	2	97.98/	65	34.34/
	(48/10)	11.31	(31/11)	13.86	(57/7)	6.08	(0/2)	0.23	(54/11)	97.54
NA11843	55	45.54/	37	63.37/	62	38.61/	1	99.01/	52	48.51/
	(48/7)	11.92	(26/11)	15.86	(54/8)	5.49	(0/1)	0.05	(52/0)	47.60
NA10847	58	38.95/	37	61.05/	63	33.68/	3	96.84/	52	45.26/
	(55/3)	11.15	(36/1)	16.24	(63/0)	6.06	(1/2)	0.5	(52/0)	92.17
NA06984	64	42.86/	46	58.93/	56	50.00/	4	96.43/	55	50.89/
	(51/13)	12.31	(32/14)	14.36	(56/0)	5.76	(0/4)	0.03	(53/2)	65.75

**Table 3:** CNV detection performances using 10 real data sets. A different germline sample (NA19152) was used as a control data set.

sample ID	ExCNVSS		ExCNVSS_noRatio		Excavator		Contra		ExomeCNV	
	Correctly detected instances (gain/loss)	FNR/FPR (%)	Correctly detected instances (gain/loss)	FNR/FPR (%)	Correctly detected instances (gain/loss)	FNR/FPR (%)	Correctly detected instances (gain/loss)	FNR/FPR (%)	Correctly detected instances (gain/loss)	FNR/FPR (%)
NA12843	31	77.04/	63	53.33/	47	65.19/	11	91.85/	64	52.59/
	(3/28)	12.04	(27/36)	14.99	(0/47)	6.20	(0/11)	0.36	(17/47)	80.09
NA12842	12	88.57/	39	62.86/	1	99.05/	3	97.14/	28	73.33/
	(3/9)	11.43	(25/14)	17.03	(0/1)	4.93	(0/3)	0.32	(17/11)	56.75
NA12748	18	82.18/	36	64.36/	1	99.01/	1	99.01/	32	68.32/
	(4/14)	11.25	(24/12)	14.47	(0/1)	6.00	(0/1)	0.26	(15/17)	70.67
NA12718	50	57.26/	76	35.04/	47	59.83/	17	85.47/	13	88.89/
	(6/44)	8.37	(31/45)	14.08	(0/47)	2.42	(0/17)	0.34	(0/13)	3.50
NA12275	22	77.55/	43	56.12/	5	94.90/	2	97.96/	9	90.82/
	(9/13)	12.13	(33/10)	13.74	(0/5)	2.70	(1/1)	0.31	(0/9)	1.85
NA12273	32	71.68/	40	64.60/	11	90.27/	17	84.96/	2	98.23/
	(0/32)	11.68	(27/13)	15.12	(0/11)	2.95	(0/17)	0.38	(0/2)	2.60
NA12272	35	64.65/	42	57.58/	16	83.84/	1	98.99/	18	81.82/
	(6/29)	12.58	(31/11)	13.86	(5/11)	2.36	(0/1)	0.18	(5/13)	1.43
NA11843	19	81.19/	37	63.37/	12	88.12/	1	99.01/	34	66.34/
	(10/9)	9.74	(26/11)	15.86	(0/12)	6.62	(0/1)	0.20	(19/15)	78.30
NA10847	13	86.32/	37	61.05/	7	92.63/	7	92.63/	9	90.53/
	(10/3)	9.58	(36/1)	16.24	(7/0)	2.56	(4/3)	0.35	(7/2)	2.03
NA06984	25	77.68/	46	58.93/	15	86.61/	13	88.39/	44	60.71/
	(2/23)	11.75	(32/14)	14.36	(0/15)	5.86	(0/13)	0.22	(15/29)	72.78
